# A Comprehensive Study of NF_3_-Based Selective Etching Processes: Application to the Fabrication of Vertically Stacked Horizontal Gate-All-around Si Nanosheet Transistors

**DOI:** 10.3390/nano14110928

**Published:** 2024-05-24

**Authors:** Xin Sun, Jiayang Li, Lewen Qian, Dawei Wang, Ziqiang Huang, Xinlong Guo, Tao Liu, Saisheng Xu, Liming Wang, Min Xu, David Wei Zhang

**Affiliations:** 1 School of Microelectronics, Fudan University, Shanghai 200433, China; 20112020027@fudan.edu.cn (X.S.); 21112020014@m.fudan.edu.cn (J.L.); lwqian21@m.fudan.edu.cn (L.Q.); 20112020120@fudan.edu.cn (D.W.); 20212020016@fudan.edu.cn (Z.H.); xlguo22@m.fudan.edu.cn (X.G.); xu_min@fudan.edu.cn (M.X.); dwzhang@fudan.edu.cn (D.W.Z.); 2 The Key Laboratory of Analog Integrated Circuits and Systems, School of Integrated Circuits, Xidian University, Xi’an 710071, China; lmwang@xidian.edu.cn; 3 Shanghai Integrated Circuit Manufacturing Innovation Center Co., Ltd., Shanghai 201202, China

**Keywords:** gate-all-around, Si nanosheet, transistors, inner spacer, channel release, isotropic dry etching, high selectivity, plasma free

## Abstract

In this paper, we demonstrate a comprehensive study of NF_3_-based selective etching processes for inner spacer formation and for channel release, enabling stacked horizontal gate-all-around Si nanosheet transistor architectures. A cyclic etching process consisting of an oxidation treatment step and an etching step is proposed and used for SiGe selective etching. The cyclic etching process exhibits a slower etching rate and higher etching selectivity compared to the direct etching process. The cycle etching process consisting of Recipe 1, which has a SiGe etching rate of 0.98 nm/cycle, is used for the cavity etch. The process achieved good interlayer uniformity of cavity depth (cavity depth ≤ 5 ± 0.3 nm), while also obtaining a near-ideal rectangular SiGe etch front shape (inner spacer shape = 0.84) and little Si loss (0.44 nm@ each side). The cycle etching process consisting of Recipe 4 with extremely high etching selectivity is used for channel release. The process realizes the channel release of nanosheets with a multi-width from 30 nm to 80 nm with little Si loss. In addition, a selective isotropic etching process using NF_3_/O_2_/Ar gas mixture is used to etch back the SiN film. The impact of the O_2_/NF_3_ ratio on the etching selectivity of SiN to Si and the surface roughness of SiN after etching is investigated. With the introduction of O_2_ into NF_3_/Ar discharge, the selectivity increases sharply, but when the ratio of O_2_/NF_3_ is up to 1.0, the selectivity tends to a constant value and the surface roughness of SiN increases rapidly. The optimal parameter is O_2_/NF_3_ = 0.5, resulting in a selectivity of 5.4 and a roughness of 0.19 nm.

## 1. Introduction

Among various proposed 3D architectures, vertically stacked horizontal gate-all-around (GAA) Si nanosheeet (SNS) transistors (GAA-SNSTs) are considered the most promising candidates to replace FinFETs in sub-3 nm technology nodes [1,2,3]. The GAA architectures have superior electrostatic control and improved suppression short-channel effects [4]. In addition, horizontal nanosheets offer design flexibility to design continuous range-width nanosheets on a single chip for simultaneous low power applications and high-performance computing. GAA-SNSTs are a natural extension of FinFETs, significantly reusing legacy integration and manufacturing knowledge [5]. However, several key process modules are distinctive to GAA-SNSTs and control device performance, including epitaxial deposition Si/SiGe super-lattice, inner spacer module, and channel release process. The inner spacer is proposed to reduce the parasitic capacitance between the gate and the source/drain, while providing a physical separation to protect the source/drain epitaxy from etch erosion during channel release [1]. The inner spacer module consists of cavity etch of the sacrificial SiGe layers followed by dielectric deposition and etch back. Channel release process is the selective removal of the sacrificial SiGe layers of the channel to obtain suspended nanosheets. 

Both the cavity etch of the inner spacer module and the channel release process require high etching selectivity of SiGe to Si and exposed dielectrics. However, the etching process must meet different requirements based on the characteristics of the process modules. The depth and shape of the inner spacer are two important criteria that have an impact on device performance [6]. For example, a shallow inner spacer will result in high parasitic capacitance between the gate and source/drain, which will affect alternating current (AC) performance. A deep inner spacer can result in a high extension resistance and worse short channel effect, which can also adversely affect device performance [7]. It is generally considered that the optimal inner spacer depth is less than 5 nm [1,8], so the etching rate of the SiGe selective etching process is more important than the etching selectivity for the inner spacer module. Correspondingly, the channel release process is more concerned with high etching selectivity to completely remove SiGe.

Various techniques have been proposed for the selective etching of SiGe to Si, including gaseous HCl thermal dry etching [9,10], plasma dry etching developed in reactive ion etching [11,12], wet etching [13,14], and chemical dry etching based on remote plasma source (RPS) [15,16]. However, only the last two etching processes are fully isotropic, which is required for the cavity etch. The HCl dry etching process involves selectivity etching SiGe by injecting a large amount of gaseous HCl together with H_2_ into the reactor under high temperature and high pressure. The etching temperature is between 600 °C and 760 °C, which introduces an undesired high thermal budget. The plasma dry etching process developed in reactive ion etching will cause plasma damage to the surface of Si nanosheets, which will seriously degrade the electrical performance of the device. A drawback is that the capillary forces inherent in wet etching may cause collapse of nanosheets, making its application to the channel release challenging [17]. In addition, the SiGe etch front obtained by wet etching is crescent-shaped, resulting in poor physical separation when applied to cavity etch [5]. Remote plasma dry etching is a purely chemical etching process using neutral plasma species. T. Salveta et al. have developed a CF_4_-based remote plasma dry etching process to achieve the isotropic and selective removal of SiGe relative to Si, giving the best selectivity factor of around 60 [17]. However, this process has a “top-down” effect, which is unacceptable for the inner spacer module. In our previous work [18], an NF_3_-based remote plasma dry etching process was proposed, which achieved the channel release of nanosheets with multiple widths from 30 nm to 80 nm with little Si loss.

In this work, we present a comprehensive study of NF_3_-based selective etching processes for the fabrication of GAA-SNSTs, including a cyclic etching process for SiGe and a continuous etching process for SiN. All processes were performed at room temperature with no excess thermal budget. The cyclic etching process consists of an oxidation treatment step using the remote O_2_/Ar plasma and an etching step using the remote NF_3_/Ar plasma. Compared to the direct etching process, the cyclic etching process has a slower etching rate and higher etching selectivity. The process parameters of the oxidation treatment step are fixed, and a series of etching recipes are obtained by tuning the parameters of NF_3_ gas flow, Ar gas flow, chamber pressure and RPS power in the SiGe selective etching process. The SiGe etching selectivity is found to be proportional to the etching rate from the results measured on the blanket wafer. The recipe with the highest etching rate has the highest etching selectivity, while the recipe with the lowest etching rate has the highest etching accuracy. Therefore, a trade-off between etching selectivity and etching rate must be made depending on the process module. The cycle etching process consisting of Recipe 1 is used for cavity etch and the cycle etching process consisting of Recipe 4 is used for channel release. In addition, an etching process using the remote NF_3_/O_2_/Ar plasma is applied to etch back the SiN film for the inner spacer module.

## 2. Materials and Methods

**Figure 1:**
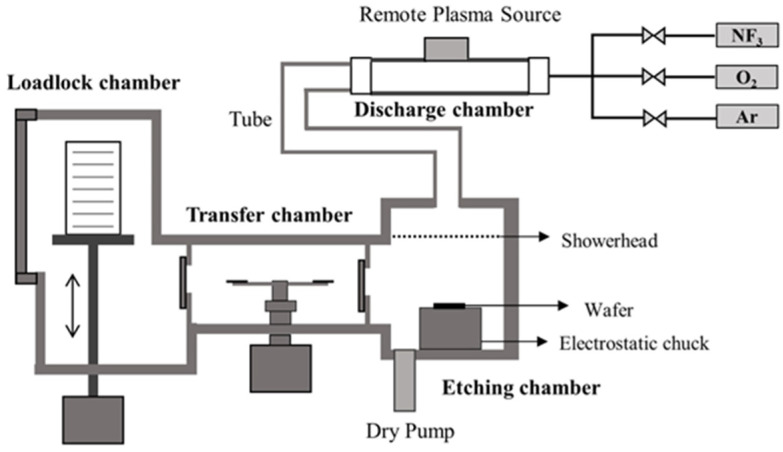
Schematic diagram of the RPS etcher.

The etching experiments were performed in a 200 mm etching platform consisting of several reactors interconnected by a vacuum transfer chamber: an RPS reactor used to achieve the isotropic and selective removal of the SiGe and three atomic layer deposition (ALD) reactors used to deposit HfO_2_, TiN, and SiN films, respectively. Schematic diagram of the RPS etcher is shown in Figure 1. The RPS is an inductively coupled plasma (ICP) source operating at 13.56 MHz. In the RPS reactor, the substrate was located on an electrostatic chuck, and the temperature was maintained at 25 °C for all the experiments. The substrate was exposed only to reactive neutrals, allowing isotropic and chemical-type etching. The pressure in the working chamber was set at 8 Torr during both the etching and oxidation processes. The RPS power was set to 2000 W or 2300 W.

The film materials used to study the SiGe selective etching process on the blanket wafer include single-layer SiGe deposited on the Si substrate using a reduced-pressure chemical vapor deposition (RPCVD) apparatus and SiN and SiO_2_ deposited on the Si substrate using a plasma-enhanced chemical vapor deposition (PECVD) apparatus. The single-layer SiGe wafer was used to obtain the SiGe etching rate, where the SiGe layer thickness was 120 nm. In addition, the SOI wafer was used to measure the Si etching rate, consisting of a 12 nm Si layer, a 25 nm SiO_2_ layer, and a Si substrate. All of the above film materials were cut into 2 × 2 cm coupon wafers. After removing the native oxide of the samples with 1% hydrofluoric acid (HF), the samples were simultaneously fixed on an 8-inch carrier wafer and transferred to the RPS reactor for etching. The etching amount of the respective materials was obtained by measuring the thickness of the film materials before and after the etching process. To investigate the SiGe selective etching process on Si/SiGe multilayer structures, three cycles of the Si/SiGe multilayer were deposited on the Si substrate using RPCVD equipment, where the Si and SiGe layers had a thickness of approximately 9 nm. The Si/SiGe super-lattice and single-layer SiGe wafers used in this work were commercially available with a Ge content of 30% in the SiGe layers. 

**Figure 2:**
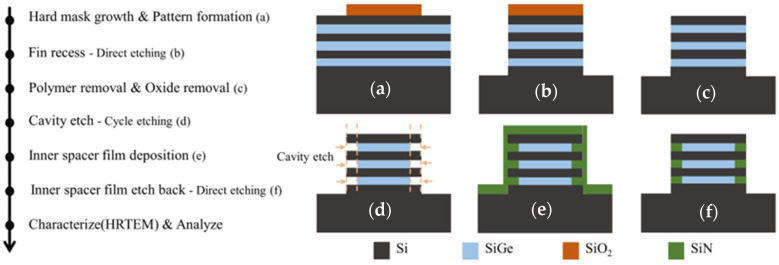
A simplified process flow and schematic diagram of inner spacer formation. (**a**) Hard mask growth and pattern formation. (**b**) Fin recess. (**c**) Polymer removal and oxide removal. (**d**) SiGe cavity etch for defining depth of the inner spacer. (**e**) Inner spacer film deposition. (**f**) Inner spacer film etch back.

**Figure 3:**
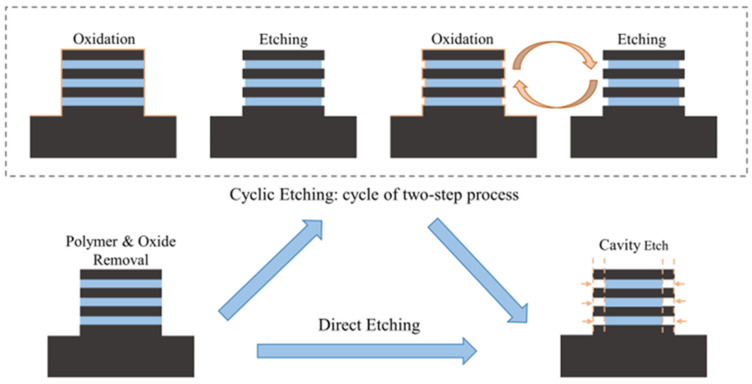
Schematic diagram of cavity etch using the direct etching process and the cyclic etching process.

A simplified process flow and schematic diagram of inner spacer formation is shown in Figure 2. First, a SiO_2_ hard mask was grown on the Si/SiGe superlattice wafer, and the designed test pattern was transferred to the hard mask (Figure 2a). The Si/SiGe super-lattice wafer was recessed by anisotropic etching (Figure 2b). Then, an in-situ oxygen (O_2_) plasma treatment process was performed to remove the fluorocarbon polymer layer on the Si/SiGe fin sidewalls. The oxide layer on the Si/SiGe fin sidewalls was then removed by soaking in a 1% HF solution (Figure 2c). Subsequently, the sacrificial SiGe layers were selectively etched by isotropic etching to form SiGe cavities that define the depth and shape of the inner spacer (Figure 2d). A SiN film was deposited by ALD technology to fill the cavities (Figure 2e). Finally, isotropic etching was used to complete the etch back of the SiN film, forming the inner spacer aligned with the entrance of the NSs (Figure 2f). The cross section of the Si/SiGe stacked multilayer was analyzed by a high-resolution transmission electron microscope (HRTEM). The process flow and the test pattern for the channel release used in this work have already been described elsewhere [18]. Figure 3 shows a schematic diagram of cavity etch using the direct etching process and the cyclic etching process.

The metal oxide semiconductor (MOS) capacitors were fabricated using the following process. P-type (100) Si substrates with an 8–12 Ω·cm were cleaned using a standard Radio Corporation of America method. After removing the oxide layer, the samples were loaded into 60 °C SC1 solution (2 NH_4_OH:5 H_2_O_2_:200 H_2_O) to grow chemical oxide. Approximately 3 nm hafnium oxide (HfO_2_) films were deposited on Si substrates at 280 °C by the ALD system using Tetrakis(dimethylamino)hafnium (TDMAH) and H_2_O as the source. Samples were processed in a rapid thermal annealing (RTA) system at 500 °C in N_2_ ambient for 1 min. Then, 100 nm TiN metal gate was deposited on the HfO_2_ film by the ALD system. Al was deposited on the backside to reduce the contact and series resistance of the samples. MOS capacitors were annealed in the forming gas (95% N_2_/5% H_2_) at 400℃ for 30 min. The equivalent-oxide thickness (EOT) and interface state density (D_it_) of the MOS capacitors were extracted from the capacitance–voltage (C–V) curves. The leakage current density was extracted from the current–voltage (I–V) curves.

An ellipsometer (J.A. Woollam Vase, J.A. Woollam, Lincoln, NE, USA) was used to measure the thickness of the film materials. Atomic force microscope (AFM, Park NX10, Park Systems, Suwon, Republic of Korea) measurements were carried out in tapping mode to investigate the surface morphology of the film materials. The AFM images shown in this work were 5 μm × 5 μm scans with a resolution of 256 points × 256 lines. HRTEM (Thermo Fisher Talos-F200X, Thermo Fisher Scientific Inc., Carlsbad, CA, USA) was used to obtain the amount of lateral SiGe etching, Si loss, and etched shape in Si/SiGe multilayer structures. The C–V characteristics and the I–V characteristics were measured using a Keithley 4200 semiconductor parameter analyzer (Keithley Inc., Cleveland, OH, USA) at room temperature.

## 3. Results and Discussion

**Figure 4:**
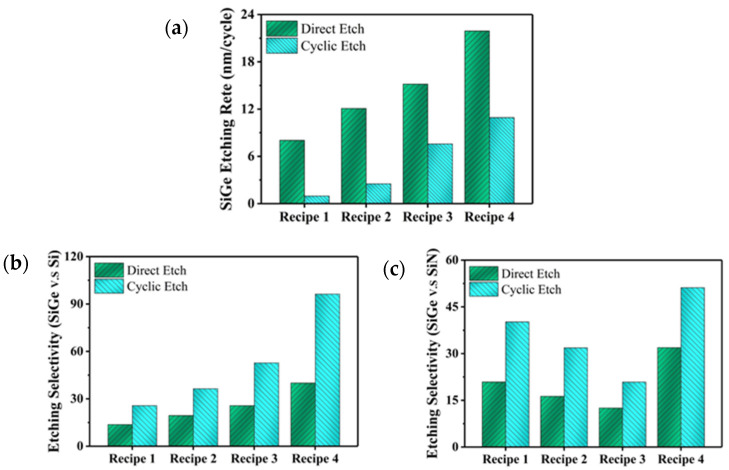
The etching characteristics of the direct etching process and the cyclic etching process consisting of different etching recipes. (**a**) SiGe etching rate. (**b**) Etching selectivity of SiGe to Si. (**c**) Etching selectivity of SiGe to SiN.

**Figure 5:**
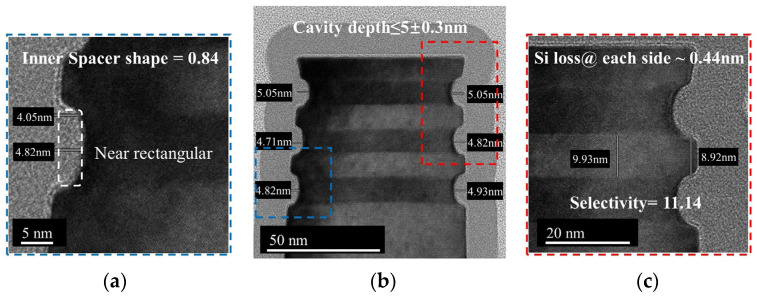
Cross-sectional TEM images of the Si/SiGe multilayer structure obtained by cyclic etching consisting of Recipe 1 with 5 cycles. (**a**) The SiGe etch front shape. (**b**) The interlayer uniformity of cavity depth. (**c**) The Si loss of NS.

**Figure 6:**
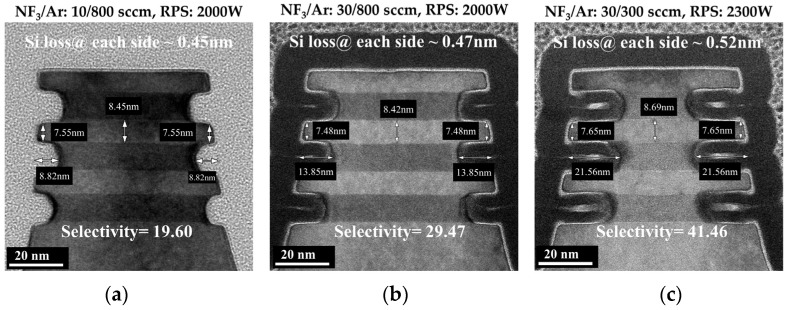
Cross-sectional TEM images of the Si/SiGe multilayer structure obtained by the cyclic etching of (**a**) 5 cycles consisting of Recipe 1, (**b**) 5 cycles consisting of Recipe 3 and (**c**) 2 cycles consisting of Recipe 4.

The SiGe selective etching process proposed in this paper is isotropic in nature. The results indicate that the SiGe etching rates obtained on the blanket wafer are essentially equivalent to those obtained on the Si/SiGe multilayer. The etching characteristics of the direct etching process and the cyclic etching process consisting of different etching recipes are shown in Figure 4. The NF_3_ exposure time of the etching step is fixed at 6 s in one cycle. The cyclic etching process with oxidation treatment achieves a lower SiGe etching rate and higher etching selectivity of SiGe to Si and SiN compared to the direct etching process. Following the oxidation treatment, an oxide layer is formed on the surfaces of Si, SiN, and SiGe, which suppresses the etching of these materials in the subsequent etching step. The inhibition of the oxides formed on Si and SiN surfaces is greater than that of the oxide formed on SiGe surface. This may be due to the weaker G-O bond compared to the Si-O bond. The bond dissociation energies of SiO_2_ and GeO_2_ have been reported to be 800 and 659 kJ/mol, respectively [19]. The SiGe selective etching process based on the RPS is a purely chemical etching without ion bombardment. The etching is primarily controlled by the composition of the gas mixture, but other parameters can also be used to tune the process, such as gas flow and excitation power. The flows of NF_3_ and Ar affect the density of reactive F atoms in the etching chamber, where NF_3_ flow is proportional to the density of F atoms and Ar flow is inversely proportional to the density of F atoms. The dissociation of NF_3_ can be affected by RPS power, which in turn can influence the etching characteristics of the process. Furthermore, the properties of the SiGe material itself exert an influence on the etching process, such as the thickness of the SiGe layer. According to previous works [12,15], the SiGe etching rate is proportional to the thickness of SiGe layer in the Si/SiGe multilayer structures. The thickness of SiGe in the Si/SiGe superlattice wafers used in this work is 10 nm, and the effect of SiGe thickness on the selective etching process will be investigated in future work. In this work, Recipe 1, Recipe 2, and Recipe 3 increased the NF_3_ gas flow in turn, while other parameters remain unchanged. Figure 4 shows a positive correlation between the SiGe etching rate and the selectivity of SiGe to Si with the NF_3_ gas flow, while a negative correlation is observed between the selectivity of SiGe to SiN and the NF_3_ gas flow. In the NF_3_-based remote plasma etching process, the reactive F atoms react with the exposed material [20,21]. As the NF_3_ gas flow increases, the density of reactive F atoms in the reactor increases, which increases the etching rate of both materials. Differences in the sensitivity of SiGe, Si, and SiN etching rates to the density of reactive F atoms lead to different trends in the etching selectivities of SiGe to Si and SiGe to SiN. Compared to Recipe 3, the RPS power of Recipe 4 is increased while the Ar gas flow is reduced. The etching selectivity of SiGe to SiN is improved in Recipe 4, while the etching selectivity of SiGe to Si is further improved. The etching rate of SiO_2_ obtained under all the above etching recipes is very slow, and it can be generally considered that the proposed SiGe selective etching process can hardly etch SiO_2_. The experimental conditions are as follows:

- Process pressure: 8 Torr (for all experiments);
- Oxidation treatment: O_2_/Ar = 50/500 sccm = 0.01, RPS power: 2300 W;
- Recipe 1: NF_3_/Ar = 10/800 sccm = 0.0125, RPS power: 2000 W;
- Recipe 2: NF_3_/Ar = 20/800 sccm = 0.025, RPS power: 2000 W;
- Recipe 3: NF_3_/Ar = 30/800 sccm = 0.0375, RPS power: 2000 W;
- Recipe 4: NF_3_/Ar = 30/300 sccm = 0.1, RPS power: 2300 W;

In this paper, the target depth of the inner spacer is 5 nm, which means that the cavity etch step needs to selectively etch SiGe with a lateral etching amount of 5 nm. Since the depth of the inner spacer determines the gate length (L_G_), the variations in the lateral etching amount of SiGe will result in GAA devices with unwanted L_G_ changes. The cyclic etching process consisting of Recipe 1 has the lowest SiGe etching rate and acceptable etching selectivity, so the recipe is applied to the inner spacer module to obtain a more accurate SiGe etching amount. Cross-sectional TEM images of the Si/SiGe multilayer structure obtained by the cyclic etching consisting of Recipe 1 with five cycles are shown in Figure 5. The SiGe etch front shape produced by the cyclic etching process is closer to an ideal rectangular with an inner spacer shape = d/*p* = 0.84 (Figure 5a). The good interlayer uniformity of cavity depth (cavity depth ≤ 5 ± 0.3 nm) is shown in Figure 5b. The little Si loss of NSs (~0.44 nm@ each side) is obtained by the cyclic etching process (Figure 5c). However, the SiGe selectivity of 11.1 for Si is significantly lower than the selectivity of 25.7 on the blanket wafer. A controlled experiment is conducted where the number of cycles is increased from five to eight. The TEM image shows a SiGe etching depth of 8.82 nm, a selectivity of 19.6, and a Si loss of 0.45 nm (Figure 6a). As the number of cycles of the cyclic etching process increased, the SiGe etching depth increased linearly, but the amount of Si loss is almost constant. Thus, the selectivity of SiGe to Si increases to a value closer to that obtained on the blanket wafer. Figure 6b shows a cross-sectional TEM image of the Si/SiGe multilayer structure obtained by the cyclic etching process consisting of Recipe 2 with 5 cycles, in which the Si loss is 0.47 nm and the selectivity is 29.5. Figure 6c shows a cross-sectional TEM image of the Si/SiGe multilayer structure obtained by the cyclic etching process consisting of Recipe 4 with two cycles, in which the Si loss is 0.52 nm and the selectivity is 41.5. The selectivity of the cyclic etching process consisting of Recipe 2 and Recipe 4 is also significantly lower than that obtained on the blanket wafer. An inherent Si loss of approximately 0.5 nm is found, which is independent of the Recipe and number of cycles of the cyclic etching process. The imperfect epitaxial process results in an intermixing layer between Si and SiGe layers in the Si/SiGe multilayer structure, which causes the inherent Si loss during the subsequent SiGe selective etching process. Due to the inherent Si loss (~0.5 nm) and small SiGe etching depth (≤5 nm) in the cavity etch step, the inner spacer module requires a slow SiGe etching rate for accurate control of the SiGe etching depth rather than extremely high selectivity. Therefore, the cyclic etching process consisting of Recipe 1 is used for the cavity etch of the inner spacer module.

**Figure 7:**
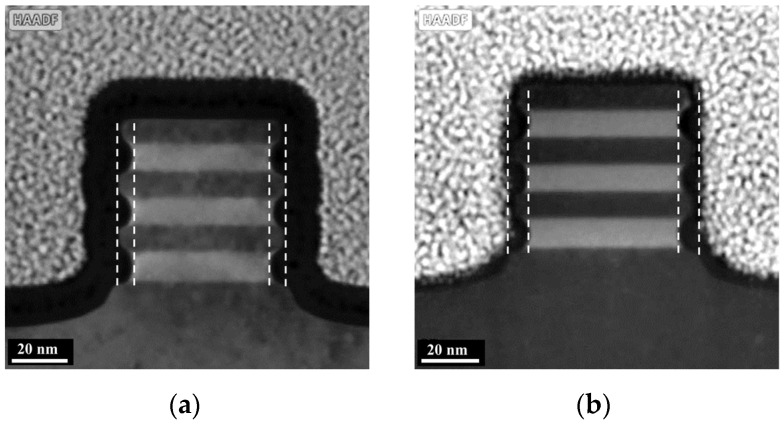
HAADF-TEM images of the Si/SiGe stacked structure after (**a**) filling the cavities with SiN and (**b**) etching back the SiN using the proposed selective etching process (O_2_/NF_3_ = 0.5).

**Figure 8:**
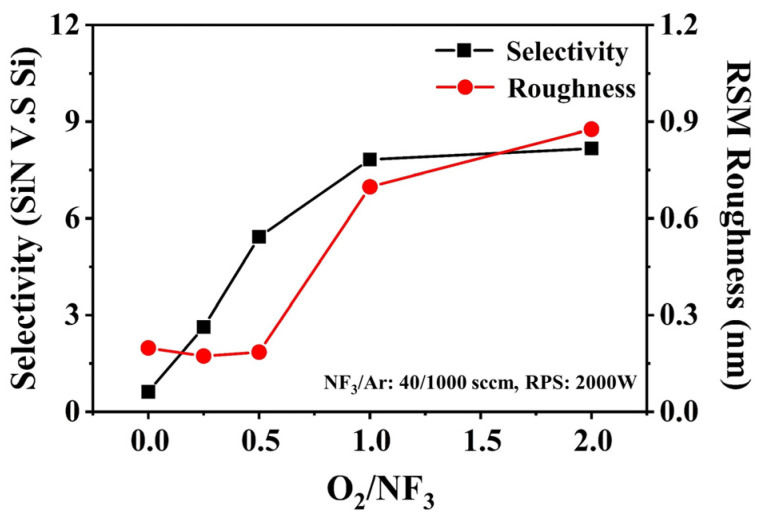
The selectivity of SiN to Si and the SiN surface roughness after the etch back process as a function of the O_2_/NF_3_ ratio.

After the cavity etch step, ALD technology was used to deposit the dielectric to fill the cavities due to its excellent gap-filling ability. For better process compatibility, SiN with high etching resistance and high density was chosen as the cavity filling material in this work. A high-angle angular dark-field TEM (HAADF-TEM) image of the Si/SiGe multilayer structure after filling the cavities with SiN is shown in Figure 7a. It can be seen that the SiN is uniformly filled into the cavities without pinholing and seaming. The final step of the inner spacer module is the dielectric etch back, which removes excess SiN through a selective etching process to form the inner spacer. Li et al. have proposed an anisotropic etching process for SiN etch back, which requires extremely high anisotropic properties and selectivity of SiN to other exposed materials [12]. In previous publications, the selective etching of SiN to Si and SiO_2_ in remote NF_3_ discharges with O_2_ addition has been studied [20,21,22,23]. It was found that the etching rate of SiN and the selectivity of SiN to Si were greatly enhanced when O_2_ was added to the NF_3_ discharge. In this work, a selective isotropic etching process for SiN etch back based on the RPS reactor using NF_3_/O_2_/Ar gas mixture is investigated. The presence of Ar in the gas mixture can enhance the dissociation rate of O_2_, thereby increasing the number of reactive nitric oxide (NO) and reactive O atoms available for the reaction. This can improve the etching selectivity of SiN to Si. (The maximum flow capacity of the mass flow controller in our etcher is 1000 sccm.) The NF_3_ flow rate is a crucial factor influencing the SiN etching rate. To achieve both high etching precision and mass production, the SiN etching rate is controlled at 0.2–1.0 nm/s. Therefore, the flow rates of NF_3_ and Ar are fixed at 40 sccm and 1000 sccm, respectively, for all experiments, while varying amounts of O_2_ are added. The effect of the O_2_/NF_3_ ratio on the etch back process is studied on the blanket wafer. Figure 8 shows the selectivity of SiN to Si and the SiN surface roughness after the etch back process as a function of the O_2_/NF_3_ ratio. When there is no O_2_ in the gas mixture, the etching selectivity of SiN to Si is less than 1, and the selectivity can be greatly improved by introducing O_2_. The introduction of O_2_ to the NF_3_ discharge produces reactive NO and reactive O atoms in the reactor. The reactive NO increases the rate of F migration on the SiN surface, while the reactive O atom produces a passivating oxide layer on the surface of Si during the etching process. This improves the etching selectivity of SiN to Si. As the O_2_ flow is further increased (up to O_2_/NF_3_ = 1.0), the selectivity tends to a constant value, and another problem is that the surface roughness of the SiN increases sharply after the etch back process. In the afterglow of NF_3_/O_2_/Ar discharges, the NO density initially increases with the addition of O_2_ and subsequently remains at a fairly constant level [24]. We speculate that when the O_2_/NF_3_ ratio is greater than 0.5, more reactive O atoms are generated in the reactor and attached to the surface site of SiN, causing the heterogeneous oxidation of the SiN surface during the etch back process. Compared to the oxidized position, the SiN etching rate at the non-oxidized position is faster, so the surface roughness of the SiN increases. Another possible reason for the increase in SiN surface roughness is the generation of solid by-products, such as (NH_4_)_2_SiF_6_ (ammonium fluorosilicate), on the SiN surface during the etching process. This paper does not discuss the etching selectivity of SiN to SiO_2_ due to the significantly smaller etching rate of SiO_2_ (less than 1 nm/min) compared to SiN. The optimal parameter is O_2_/NF_3_ = 0.5, resulting in a selectivity of 5.4 and a roughness of 0.19 nm. A HAADF-TEM image of the Si/SiGe multilayer structure after etching back the SiN using the proposed selective etching process (O_2_/NF_3_ = 0.5) is shown in Figure 7b.

**Figure 9:**
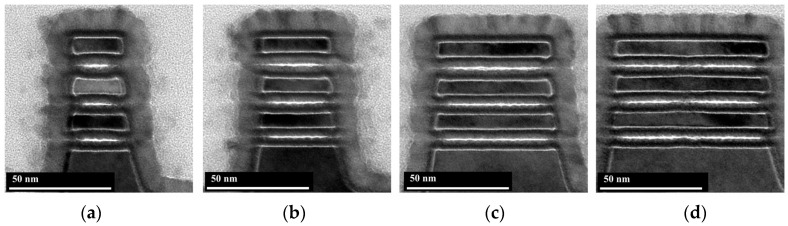
Cross-sectional TEM images of stacked NSs with multiple widths obtained by the cyclic etching process consisting of Recipe 4. (**a**) 30 nm; (**b**) 40 nm; (**c**) 60 nm; (**d**) 80 nm.

**Figure 10:**
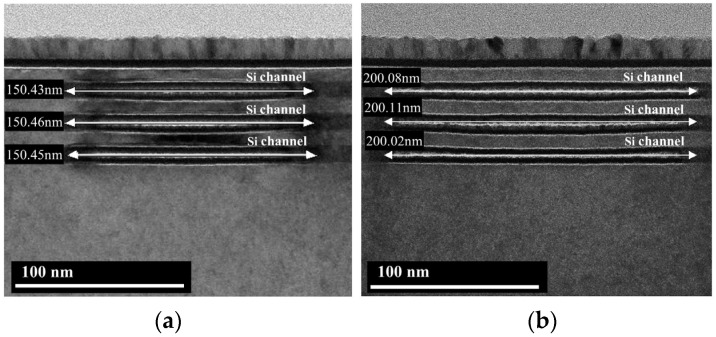
Cross-sectional TEM images of the NSs with a length of (**a**) 150 nm and (**b**) 200 nm, obtained by cutting along the direction of the channel length.

To achieve the co-optimization of performance and power, the simultaneous channel release of multi-width NSs is required, which requires the channel release process to have an extremely high etching selectivity of SiGe to Si. In our previous work [18], we proposed a novel multi-step etching process that could realize the channel release of NSs with multiple widths from 30 nm to 80 nm with little Si loss [18]. The oxidation treatment in the multi-step etching process is similar to natural oxidation and takes more than 20 min. In comparison, the oxidation treatment in the cyclic etching process proposed in this paper utilizes an O_2_/Ar remote plasma, which offers a higher oxidation efficiency and a shorter process time of only 1 min. Figure 9 shows cross-sectional TEM images of stacked NSs with multiple widths obtained by the cyclic etching process consisting of Recipe 4. The NSs cross sections tend to be ideally rectangular with little Si loss at the entrance of the NSs. To better analyze whether the NSs are deformed in the stacking direction after the channel release process, samples with a width of 60 nm are cut along the direction of the channel length. Cross-sectional TEM images show that the NSs with a length of 150 nm are equally spaced in the stacking direction, indicating almost no deformation, and that the NSs with a length of 200 nm are only slightly deformed (Figure 10). In our previous work [18], we investigated the effect of the source/drain compressive stress on the mechanical stability of stacked Si NSs during the process of channel release. Compared with the traditional channel release process, which completes the etching of the sacrificial layer by one-step etching, the multi-step etching process can solve the problem of severe deformation of the NSs by gradually releasing the stress. The cyclic etching process proposed in this work, when applied to channel release, also achieves the removal of the SiGe sacrificial layer by multi-step etching. As a result, the NSs with a length of 150 nm undergo almost no deformation. The longer the length of the NS, the more likely it is to deform under the same stress. Therefore, the NSs with a length of 200 nm deformed slightly.

**Figure 11:**
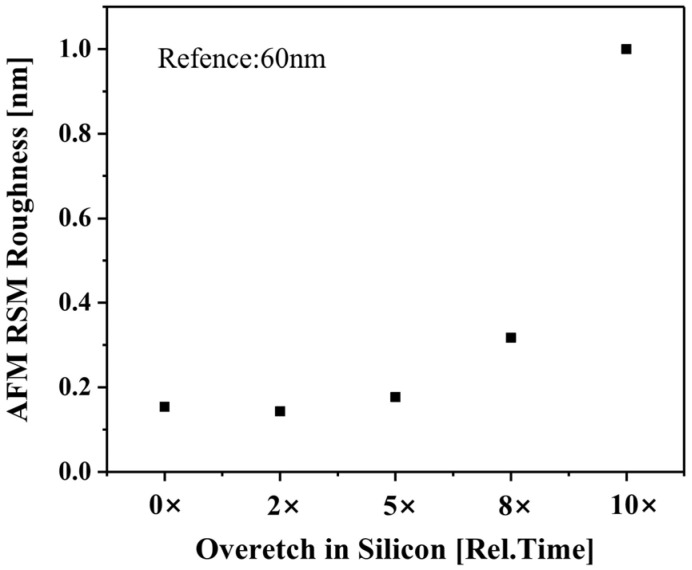
Si surface roughness post channel release exposed to increased overetch.

Both increased surface roughness and etching damage of Si NSs can lead to mobility degradation, which reduces the performance of GAAFET devices. The size of the NSs is too small and in a suspended state, making it impossible to measure the roughness of the NSs directly with AFM. Given that the thickness of the Si layer of the SOI wafer is comparable to that of the NSs, and both are monocrystalline Si, the SOI wafer was used to measure the surface roughness of Si after the channel release process. As shown in Figure 11, the proposed cyclic etching process has no impact on Si surface roughness, which has sub-monolayer roughness even for 8× the normal etching amount (30 nm@ each side). To evaluate the potential etching damage to the surface of Si NSs caused by the proposed cyclic etching process, we selected D_it_ and leakage current density (@V_FB_-1V) as the primary assessment criteria. After correction of the measurement data for parasitic effects, D_it_ was extracted by the optimized conductance method proposed by W.A. Will [25]. The reference sample was directly utilized to fabricate the MOSCAPs using the process flow described above. In contrast, the CR sample underwent a pre-treatment involving the cyclic etching process prior to the MOSCAPs fabrication, intended to emulate the channel release in the process flow of GAA. For each sample, over 20 MOSCAPs were measured to ensure the stability of the electrical characteristics. The MOSCAPs fabricated on the CR sample exhibited an average D_it_ and a leakage current density of 2.32 × 10^−11^ cm^2^ eV^−1^ and 1.43 × 10^−4^ A/cm^2^, respectively. Correspondingly, the MOSCAPs fabricated on the reference sample had an average D_it_ and a leakage current density of 2.26 × 10^−11^ cm^2^ eV^−1^ and 1.36 × 10^−4^ A/cm^2^, respectively. The results indicate that the cyclic etching process proposed in this work for channel release is free of etching damage to the Si NSs.

## 4. Conclusions

Inner spacer formation and channel release are two critical modules within GAA device integration, playing a pivotal role in determining the performance, yield, and reliability of device. This paper presents a comprehensive study of NF_3_-based selective etching processes, which enable stacked horizontal GAA-SNST architectures. We propose a cyclic etching process for SiGe selective etching, consisting of an oxidation treatment step and an etching step. The cyclic etching process exhibits a slower etch rate and higher etch selectivity compared to the direct etching process. The cycle etching process consisting of Recipe 1 is used for the cavity etch, which achieves good interlayer uniformity of cavity depth, a near-ideal rectangular SiGe etch front shape and little Si loss of NSs. An intrinsic Si loss about 0.5 nm independent of the etching process is found in the process of cavity etch, which is caused by the imperfect epitaxy of Si/SiGe superlattice wafer. The cycle etching process consisting of Recipe 4 is used for channel release, which realizes the channel release of nanosheets with a multi-width from 30 nm to 80 nm with little Si loss. We demonstrate that the proposed cyclic etching process is free of etching damage to the Si NSs and had no effect on the Si surface roughness. In addition, a selective isotropic etching process using an NF_3_/O_2_/Ar gas mixture is investigated for etching back the SiN film. It is found that the O_2_/NF_3_ ratio has a significant effect on the etching selectivity of SiN to Si and surface roughness of SiN after etching. The optimal parameter of O_2_/NF_3_ = 0.5 achieves moderate etch selectivity (5.4) and no significant increase in surface roughness (0.19 nm).

## Data Availability

The data presented in this study are available on request from the corresponding authors.
